# Systemic brain dissemination of glioblastoma requires transdifferentiation into endothelial-like cells via TGF-β-ALK1-Smad1/5 signaling

**DOI:** 10.1016/j.neo.2024.101110

**Published:** 2024-12-25

**Authors:** Thomas M.B. Ware, Adilson Fonseca Teixeira, Josephine Iaria, Rodney B. Luwor, Hong-Jian Zhu

**Affiliations:** a Department of Surgery, The Royal Melbourne Hospital, The University of Melbourne, Parkville 3050 Victoria, Australia; b Huagene Institute, Kecheng Science and Technology Park, Pukou District, Nanjing 211806, Jiangsu, PR China; c Fiona Elsey Cancer Research Institute, Ballarat, Victoria 3350, Australia; d Federation University, Ballarat, Victoria 3350, Australia; e Lead contact

**Keywords:** Glioblastoma, Angiogenesis, Phenotypic plasticity, TGF-β signaling, Targeted therapy

## Abstract

Glioblastoma is the most aggressive type of brain cancer, but treatment improvements for glioblastoma patients remain stagnated for over 20 years. This is despite the large number of clinical trials that have attempted to replicate the success of therapeutics developed for other cancer types. This discrepancy highlights the urgent need to decipher the unique biology of glioblastomas. Here, we show that glioblastoma tumour cells are highly plastic, integrating into blood vessel walls to disseminate throughout the brain. This relies on the transdifferentiation of glioblastoma tumor cells into endothelial-like cells in a process we termed endothelialisation. Mechanistically, in addition to TGF-β-ALK5-Smad2/3 signaling, glioblastoma tumour cells also activate TGF-β-ALK1-Smad1/5 signaling – a mechanism previously thought to be limited to endothelial cells. Consequently, therapeutic targeting of TGF-β-ALK1-Smad1/5 activity impaired endothelialisation-driven glioblastoma progression. This study identifies a previously unknown component of glioblastoma biology and establishes a therapeutic approach to reduce the progression of this disease.

## Introduction

Glioblastoma is a WHO grade IV primary brain tumour and one of the most debilitating forms of human cancers [1]. Despite an aggressive standard of care, consisting of maximal surgical resection, radiotherapy and chemotherapy, glioblastoma inevitably recurs [2,3]. This leads to a dismal median overall survival of 12-14 months, which has not been significantly improved for nearly 50 years [3,4]. The stagnation in glioblastoma survival has led to a plethora of new research that advanced our understanding of this cancer type and contributed for the development of new targeted therapies [[5], [6], [7]].

The development of angiogenesis inhibitors, for instance, was a major breakthrough in the field of targeted therapies and their use was once considered the most promising treatment for glioblastomas due to the extensive and unique vascularity observed in this type of cancer [8]. However, after nearly two decades of research, clinical trials investigating the use of such inhibitors have failed to demonstrate relevant overall survival benefit in glioblastoma [9,10]. This observation suggests that the original assumptions about the establishment and expansion of blood vessels in glioblastoma may have been misappropriated [3].

Close examination of the glioblastoma vasculature reveals abnormalities in blood vessel structures, which are often dilated with large gaps in the endothelium walls [11]. Interestingly, the TGF-β signalling inhibitor galunisertib has been shown to attenuate such abnormalities in a glioblastoma xenograft model [12]. Increased TGF-β signaling activity drives cancer progression and is associated with poor prognosis for glioblastoma patients [13,14]. Activation of the TGF-β signalling in glioblastoma cells is known to increase the phosphorylation and activation of the intracellular effectors Smad2/3 [15]. If not inhibited by endogenous (e.g., Smad6 and Smad7) or pharmacological (e.g., galunisertib or SB-431542) agents, increased TGF-β-Smad2/3 signaling activity in cancer cells induce cancer cell migration and invasion [13]. Strikingly, glioblastoma tumour cells have also been described to increase Smad1/5 phosphorylation in response to TGF-β, an effect previously thought to be specific to endothelial cells [[16], [17], [18]]. Although activation of TGF-β-Smad1/5 signaling in endothelial cells promotes angiogenesis [18], the precise role played by this molecular pathway in glioblastoma cells remains unknown.

Here, we hypothesize that TGF-β-Smad1/5 signaling activation drives glioblastoma progression by regulating the development and expansion of the abnormal vasculature observed in glioblastomas. Our results show that glioblastoma cells incorporate into tumor blood vessels to promote systemic brain dissemination. Demonstrating the role played by TGF-β-Smad1/5 signaling in this process, knockdown of the TGF-β type I receptor ALK1 or overexpression of the inhibitory Smad6 reduced endothelialisation and impaired glioblastoma progression. Thus, our findings open a new avenue to improve the survival of glioblastoma patients as reduced endothelialisation may be achieved by therapies targeting TGF-β-Smad1/5 signaling.

## Results

### Glioblastoma tumor cells occupy abnormal gaps in the endothelium of human tumour sections and form blood vessel-like lattice structures ***in vitro***

**Fig. 1:**
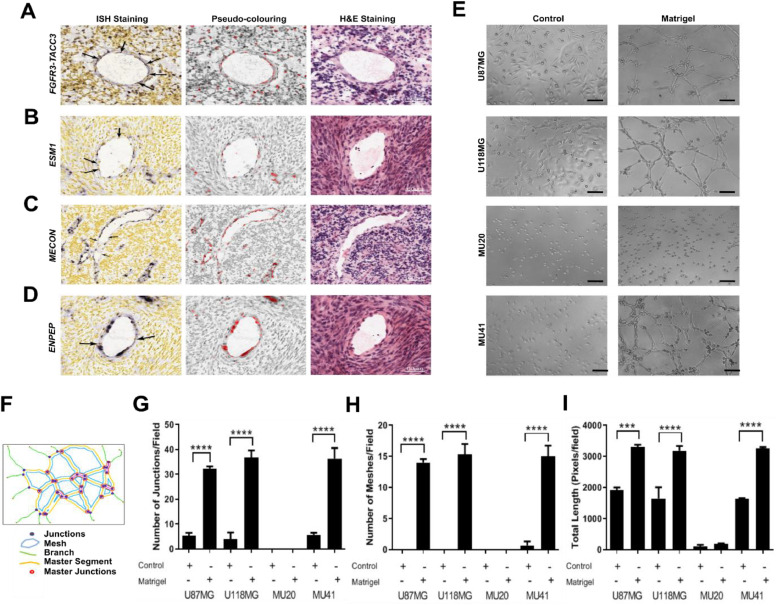
Glioblastoma tumor cells occupy abnormal gaps in the endothelium of human tumour sections and form blood vessel-like lattice structures *in vitro. In situ* hybridisation (ISH) and H&E images were obtained from the Ivy Glioblastoma Atlas Project (Ivy GAP). Images are representative of a single patient with confirmed *FGFR3-TACC3* fusion gene. A tumour blood vessel with (A) *FGFR3-TACC3*, (B) *ESM1*, (C) *MECON*, and (D) *ENPEP* positive ISH staining. Arrows in the left panels indicate positive staining within the endothelium. Middle panel shows the pseudo-colouring for cells positively stained for the target nucleic acid. Right panel shows the nearest H&E section. (E) Morphological analysis of glioblastoma cell lines seeded onto empty (control) or matrigel-coated wells. Scale bar: 50 μm. (F) Schematic illustration representation lattice-related parameters quantified *in vitro* by using ImageJ. (G) Number of junctions, (H) number of meshes, and (I) cell total length were quantified and normalized per field. Results represent mean ± SEM relative to three independent experiments (n=3). ***p < 0.001, ****p < 0.0001.

To examine the vasculature of human glioblastoma cases, we obtained primary glioblastoma sections from the publicly available Ivy Glioblastoma Atlas Project (Ivy GAP) [19]. This atlas provides a resource of *in situ* hybridisation (ISH) and adjacent hematoxylin and eosin (H&E)-stained tissue sections. Blood vessels were determined using H&E-stained adjacent tissue sections (Fig. 1 and Fig. S1) and *FGFR3-TACC3* was used as a tumour marker (Fig. 1A and S1A). *FGFR3-TACC3* is a tumour specific fusion-gene, which occurs in 1-6 % of glioblastoma patients and is present within two Ivy GAP patients [20]. Because ISH staining for the prototypical endothelial marker CD31 (*PECAM1*) is not available in the Ivy GAP, analysis of glioblastoma vasculature considered *ESM1, MECOM,* and *ENPEP*; genes known to be enriched in endothelial cells [21,22] (Fig. 1B-1D and S1B-S1D). As expected, *ESM1* expression was observed in almost all blood vessels (Fig. 1B and Fig. S1B). However, some of the cells lining blood vessels displayed no detectable staining for *ESM1* (Fig. 1B and Fig. S1B). Similarly, *MECOM* and *ENPEP* were also highly expressed within the tumour vasculature, although unstained cells were still present within blood vessels suggesting irregularities in the vascular makeup (Fig. 1C&D and Fig. S1C&D). Remarkably, *FGFR3-TACC3* positive cells not only surrounded blood vessels but were rather extensively incorporated into the blood vessels of samples from both patients examined (Fig. 1A and Fig. S1A).

To validate that these irregularities are not specific to *FGFR3-TACC3* positive glioblastomas, we analysed other patient sections within the Ivy GAP dataset (Fig. S2). Gaps in the staining of *ESM1, MECOM* and *ENPEP* were also found within the endothelium of patients negative for the *FGFR3-TACC3* (Fig. S2A-S2C). Some patients, however, displayed complete staining of these endothelial markers, with no gaps or unstained nuclei present (Fig. S2A-S2C). To confirm these findings, we examined expression of *EPAS1*, which was not available for *FGFR3-TACC3*-positive patients and is highly enriched in endothelial cells of the tumour vasculature [23]. *EPAS1* was found to be upregulated in most patient samples examined, yet unstained nuclei were still detected in most of these sections (Fig. S2D). Although caution should be used in the interpretation of *EPAS1* as an endothelial marker since it can also be expressed by glioblastoma cells [24], lack of staining supports irregularities in the blood vessel structure. Lastly, we looked at *GFAP* as a marker of tumour cells [25], finding that *GFAP*-positive cells were incorporated into all patients with available tissue sections for this marker (Fig. S1E and Fig. S2E). Overall analysis of endothelial-enriched markers revealed gaps in the tumour vasculature ranging from 60.0 %-87.5 % of all cases, while *GFAP* staining was only available in 7 patients and was detected in the tumour vasculature of all patients (Table S1). Together these results provide evidence of specific incorporation of glioblastoma tumour cells into the blood vessels of human glioblastoma patients and the inherent biological abnormality of the tumour vasculature needs to be further characterised.

In order to investigate how glioblastoma tumour cells could integrate into the tumour vasculature, we firstly assessed and compared the lattice formation properties of glioblastoma cell lines using matrigel as a basement membrane surrogate to mimic tumorigenic conditions. Compared with cells cultured in uncoated wells, U87MG, U118MG and MU41 cell lines seeded in matrigel-coated wells showed obvious lattice formation (Fig. 1E), recapitulating endothelial cell behaviour [26]. MU20 cells, however, did not form any lattice structures, presenting a spherical and undifferentiated-like morphology (Fig. 1E). Additional quantification using the ImageJ macro “angiogenesis analyzer” (Fig. 1F) showed increased inter-cell connectivity for U87MG, U118MG and MU41 cell lines seeded onto matrigel as measured by an increase in the number of junctions and mesh-like structures per field (Fig. 1G-1I). Increased cell elongation was also observed in matrigel-coated wells compared to control (Fig. 1I). In contrast, MU20 cells did not show any change in inter-cell connectivity or morphology (Fig. 1G-1I). These results demonstrate that glioblastoma tumour cells are capable of phenotypically switching in endothelial-like cells; a process we termed endothelialisation.

### TGF-β activates Smad1/5 signaling to drive lattice formation in U87MG, U118MG and MU41 cells but not in MU20 cells

**Fig. 2:**
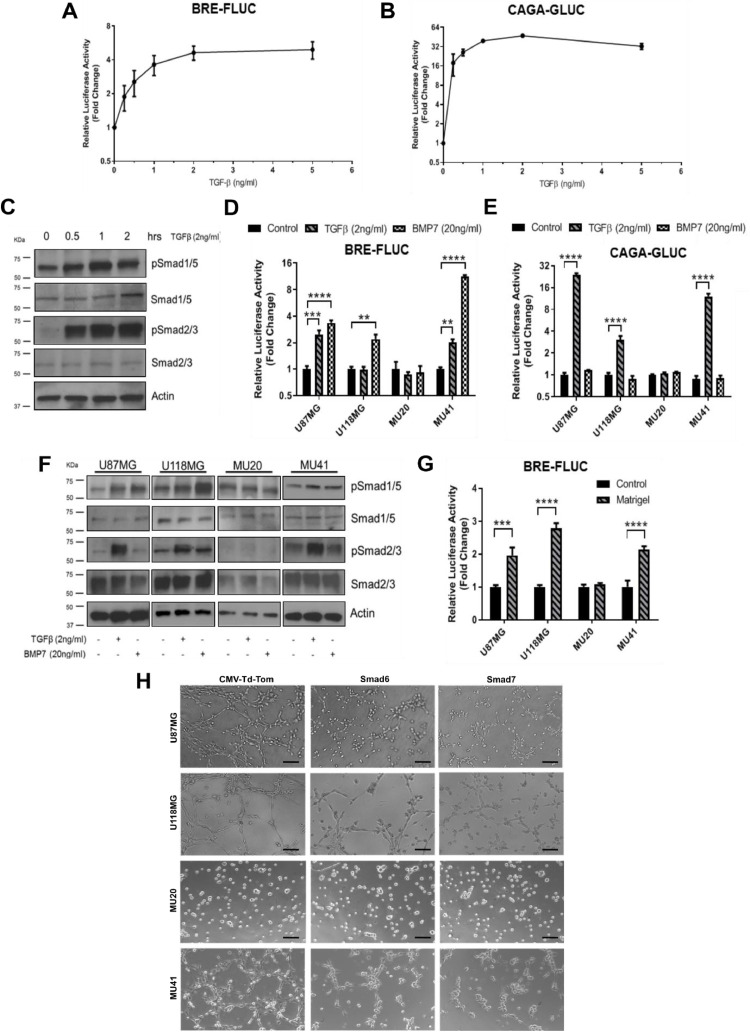
**TGF-β activates Smad1/5 signaling to drive lattice formation in U87MG, U118MG and MU41 cells but not in MU20 cells. (A-B)** U87MG glioblastoma cells were concomitantly transduced with BRE-FLUC and CAGA-GLUC. Cells were serum starved and treated with indicated concentrations of TGF-β for 6 hours before quantification of **(A)** Firefly luciferase activity and **(B)** Gaussia luciferase activity. **(C)** Phosphorylated (p)Smad1/5 and pSmad2/3 levels were analysed in U87MG cells. Cell cultures were serum starved and treated with TGF-β for indicated time periods before lysis and western blot analysis. Total Smad1/5, total Smad2/3, and Actin were used as loading controls. **(D)** Firefly luciferase activity and **(E)** Gaussia luciferase activity were quantified in glioblastoma cells as described in (A) and (B), respectively. Cells were serum starved and treated with 2 ng/mL TGF-β or 20 ng/mL BMP7 for 6 h before lysis. **(F)** pSmad1/5 and pSmad2/3 levels were analysed in glioblastoma cell lines as described in (C). Cell cultures were serum starved and treated with 2 ng/mL TGF-β or 20 ng/mL BMP7 for 1 h before lysis. **(G)** Firefly luciferase activity was quantified in glioblastoma cell lines transduced with BRE-FLUC as described in (A). Cell cultures were seeded onto control or matrigel-coated wells for 6 h before lysis. **(H)** Morphological analysis of glioblastoma cell lines transduced with *Ad-CMV-Tom* (control adenovirus), *Ad-Smad6* or *Ad-Smad7*. Forty-eight hours after transduction, cells were seeded onto uncoated or matrigel-coated wells and analysed 6 h post-seeding. Scale bar: 50 μm. Results represent mean ± SEM relative to three independent experiments (n=3). **p < 0.01, ***p < 0.001, ****p < 0.0001.

Having established endothelial cell-like mimicry in U87MG, U118MG and MU41 glioblastoma cell lines, we next sought to investigate the underlying mechanisms regulating the phenotypic plasticity in these cells. Decreased vascular abnormalities have been reported in glioblastoma xenografts treated with galunisterib, a small molecule targeting the kinase activity of the TGF-β type I receptor ALK5 [12]. Yet, TGF-β is known to activate many intracellular effectors, including the Smad2/3 (in most cell types) and Smad1/5 (previously thought to be endothelial cell-specific) [18]. Using a Smad1/5-specific promoter BRE-luciferase reporter [27,28], we confirmed TGF-β dose-dependent activation of the BRE-mediated transcription in U87MG cells (Fig. 2A). Similarly, the Smad2/3 specific promoter CAGA_12_ [ref. 27,[29], [30], [31], [32]] was also activated in a TGF-β dose-dependent manner in this cell line (Fig. 2B). Western blot analysis further confirmed activation of both TGF-β-Smad1/5 and TGF-β-Smad2/3 signalling pathways in U87MG cells treated with TGF-β (Fig. 2C). Moreover, U87MG and MU41 cells showed over 2-fold increases in BRE-luciferase reporter activity in response to 2 ng/mL TGF-β and 20 ng/mL BMP7, used as a positive control for Smad1/5 activation (Fig. 2D). Interestingly, BRE-luciferase activity remained unchanged in U118MG cells treated with TGF-β, although these cells were responsive to BMP7 (Fig. 2D). Additionally, U87MG, U118MG, and MU41 cells displayed increased CAGA_12_-luciferase reporter activity with TGF-β treatment (Fig. 2E). In contrast, MU20 cells did not respond to either TGF-β or BMP7 stimulation (Fig. 2D-2E). Western blot analysis further confirmed increased phosphorylation of Smad1/5 and Smad2/3 with TGF-β treatment in U87MG and MU41 cells, while only Smad2/3 phosphorylation was detected in U118MG cells (Fig. 2F). In turn, we could not detect increased phosphorylation of neither Smad1/5 or Smad2/3 in MU20 cells (Fig. 2F). Strikingly, U87MG, U118MG and MU41 cells seeded in matrigel-coated wells showed significant increase in BRE-luciferase reporter activity when compared with cell cultures seeded in uncoated wells (Fig. 2G). MU20 cells, however, remained unresponsive in these conditions as quantified by BRE-luciferase reporter activity (Fig. 2G).

After confirming TGF-β-Smad1/5 signalling activation in the lattice forming glioblastoma cell lines, we sought to specifically inhibit this molecular pathway to investigate its functional relevance on tumour cell endothelialisation. We utilised adenoviruses to efficiently transduce tumour cells with the specific Smad1/5 inhibitor Smad6 (*Ad-Smad6-M2* adenovirus) or the Smad1/5 and Smad2/3 inhibitor Smad7 (*Ad-Smad7-M2* adenovirus) (Fig. S3A-S3C). Using the BRE- and CAGA_12_-luciferase reporters, we confirmed the specificity and sensitivity of *Ad-Smad6* and *Ad-Smad7* viruses for the TGF-β signalling pathways. *Ad-Smad6* specifically inhibited TGF-β and BMP7 mediated activation of the BRE-luciferase reporter but did not affect CAGA_12_-luciferase reporter activity in U87MG, U118MG and MU41 cells (Fig. S4). *Ad-Smad7* also inhibited activation of the BRE-luciferase reporter in these glioblastoma cell lines for both TGF-β and BMP7 stimulation, while additionally inhibiting TGF-β mediated activation of the CAGA_12_-luciferase reporter (Fig. S4). Together these results confirm *Ad-Smad6* as a specific inhibitor of Smad1/5 signalling, while *Ad-Smad7* is both inhibitory to Smad1/5 and Smad2/3 activation in U87MG, U118MG and MU41 cells. Noteworthy, as MU20 cells were not responsive to TGF-β or BMP treatment, transduction with *Ad-Smad6* or *Ad-Smad7* did not impact BRE- or CAGA_12_-luciferase reporter activity (Fig. S4C).

**Fig. 3:**
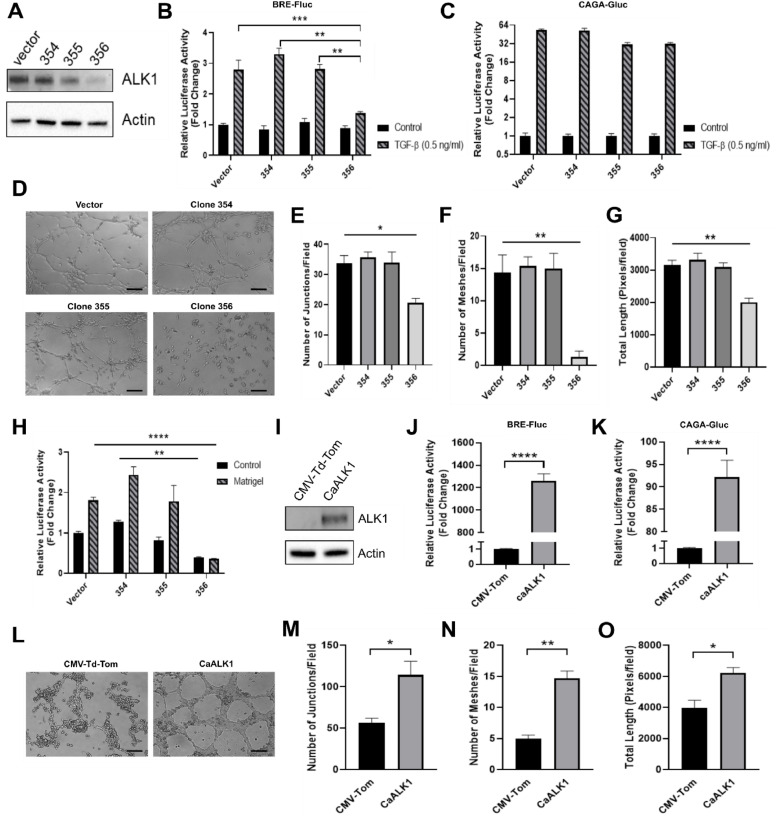
**TGF-β-induced lattice formation in glioblastoma cells requires ALK1-Smad1/5 signaling. (A)** Western blot analysis of U87MG stably transfected with vector or ALK1-shRNA 354, 355 or 356. Actin was used as loading control. Quantification of **(B)** Firefly luciferase activity and **(C)** Gaussia luciferase activity in U87MG clones transduced with BRE-Fluc and CAGA-Gluc. Cells were serum starved and treated with 2 ng/mL TGF-β for 6 h before lysis. **(D)** Morphological analysis of U87MG clones seeded onto uncoated or matrigel-coated wells for 6 h. Cells were seeded 48 h after transduction with BRE-Fluc. Scale bar: 50 μm. Images obtained for cells seeded onto uncoated wells are not shown. **(E)** Number of junctions, **(F)** number of meshes, and **(G)** cell total length were quantified and normalized per field for U87MG clones. **(H)** Firefly luciferase activity was quantified in U87MG cell culture lysates following image acquisition of lattice formation as described in (B). **(I)** Western blot analysis of ALK1 levels in MU20 cell transduced with *Ad-CMV-Td-Tom* (control adenovirus) or *Ad-caALK1*. Actin was used as loading control. **(J)** Firefly luciferase activity and **(K)** Gaussia luciferase activity were quantified in MU20 cells concomitantly transduced with BRE-Fluc and CAGA-Gluc as described in (B) and (C), respectively. *Ad-CMV-Td-Tom* and *Ad-caAK1* adenovirus were used as described in (I). **(L)** Morphological analysis of MU20 cells transduced with *Ad-CMV-Td-Tom* or *Ad-caALK1* 24 h after seeding. Cells were transduced 48 h before seeding. Scale bar: 50 μm. **(M)** Number of junctions, **(N)** number of meshes, and **(O)** cell total length were quantified and normalized per field for MU20 cells analysed in (L). Results represent mean ± SEM relative to three independent experiments (n=3). *p < 0.05, **p < 0.01, ***p < 0.001, ****p < 0.0001.

We next inquired if Smad6 and Smad7 could interfere with the formation of lattice structures. Indeed, transduction with *Ad-Smad6* and *Ad-Smad7* caused significant disruption to lattice formation in U87MG, U118MG and MU41 cells, but not MU20 cells, compared to cell cultures transduced with the control adenovirus *Ad-CMV-Tom* (Fig. 3H and S5A-S5J). MU20 cells, marked by spherical morphology even under control conditions, did demonstrate any change in cell length after *Ad-Smad6* or *Ad-Smad7* transduction (Fig. S5G).

To further validate the role played by the TGF-β-Smad1/5 signaling in lattice formation, on-target inhibition of Smad1/5 was confirmed for each treatment group following image acquisition for lattice disruption (Fig. S5K-S5N). *Ad-Smad6* and *Ad-Smad7* transduction demonstrated specific inhibition of BRE-luciferase activity compared to control uncoated wells in U87MG (Fig. S5K), U118MG (Fig. S5L) and MU41 (Fig. S5N) cell lines. MU20 cells did not demonstrate any increased BRE-luciferase activity when seeded on matrigel nor responded to *Ad-Smad6* or *Ad-Smad7* treatment (Fig. S5M).

### TGF-β-induced lattice formation in glioblastoma cells requires ALK1-Smad1/5 signaling

Because Smad1/5 can be activated by both TGF-β and BMP ligands, we sought to ascertain the contribution of TGF-β signalling to lattice formation in glioblastoma. To this aim, SB-431542 was used as a small molecule kinase inhibitor that specifically targets the TGF-β type I receptor ALK5, but not BMP type I receptor [27,30]. Treatment with 10µM SB-431542 prevented TGF-β mediated activation of both BRE- and CAGA_12_-luciferase reporter compared to vehicle treatment (Fig. S6A-S6B). Additionally, SB-431542 treatment abolished lattice formation in U87MG (Fig. S6C), preventing the formation of junctions or meshes and significantly reducing total cell length (Fig. S6D-S6F).

In endothelial cells, TGF-β-Smad1/5 signalling transduction relies on the TGF-β type I receptor ALK1 [17,33]. Although increased Smad1/5 activity has been described in glioblastoma tumour cells treated with TGF-β [16], the role played by ALK1 remained unknown. To test the function of ALK1 in Smad1/5 signalling, we used three different shRNA sequences (shRNA: 354, 355, 356) and generated stable shRNA-ALK1 knockdown in U87MG cells (Fig. 3A). Consistently, shRNA-ALK1 356 potently inhibited TGF-β-induced BRE-luciferase activity, without significantly impacting TGF-β-induced CAGA_12_-luciferase activity (Fig. 3B-3C). Furthermore, lattice formation by U87MG cells transduced with shRNA-ALK1 356 was strongly impaired (Fig. 3D-3G). Additionally, specific inhibition of BRE-luciferase activation was also confirmed in U87MG cells transduced with shRNA-ALK1 356 when seeded onto matrigel compared to vector control (Fig. 3H).

To further confirm the role of ALK1-Smad1/5 signalling in glioblastoma endothelialisation, we transduced lattice formation-incompetent MU20 cells with a constitutively active ALK1 receptor (*Ad-caALK1*) (Fig. 3I). Transduction with *Ad-caALK1* resulted in significant activation of the BRE-luciferase reporter in MU20 cells (Fig. 3J). To a lesser degree, CAGA_12_-luciferase reporter activity was also detected in these cells (Fig. 3K). Reinforcing the role played by ALK1-Smad1/5 signaling in glioblastoma cell endothelialisation, expression of caALK1 in MU20 cells induced patterned lattice formation structures (Fig. 3L). In this context, cell elongation and the number of cell junctions and lattice meshes significantly increased in the *Ad-caALK1* transduced cells compared to control (Fig. 3M-3O).

### TGF-β-Smad1/5 signaling activity drives glioblastoma tumour cell endothelialisation *in vivo*

**Fig. 4:**
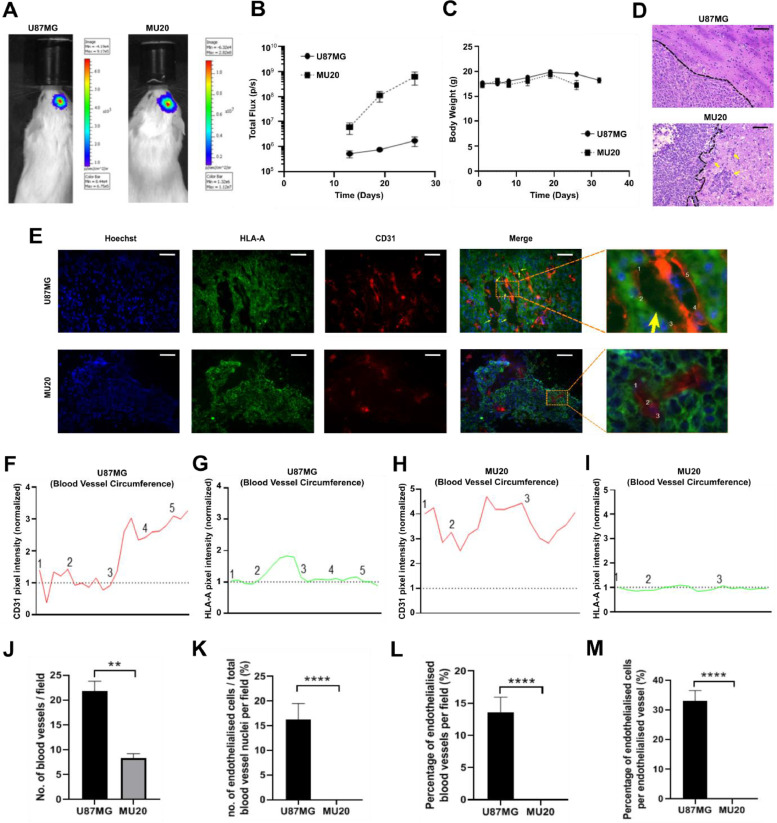
**TGF-β-Smad1/5 signaling activity drives glioblastoma tumour cell endothelialisation *in vivo*. (A)** Representative bioluminescence images and **(B)** quantification of the bioluminescence emitted by U87MG-FLUC and MU20-FLUC cells implanted into the right cortex of NOD-SCID mice. Six mice (n=6) were analysed in each group. **(C)** Body weights of mice implanted with U87MG-FLUC or MU20-FLUC cells. **(D)** Representative H&E staining of U87MG-FLUC and MU20-FLUC tumours. Arrows indicate clustering of invasive cells moving beyond the primary tumour margin. Scale bar: 50 μm. **(F)** Representative immunofluorescence staining analysis for HLA-A and CD31 in U87MG-FLUC and MU20-FLUC tumour sections. Arrows indicate CD31^−^/HLA-A^+^ vasculature staining. Scale bar: 50 μm. **(G-J)** A section of tumour vasculature analysed in (F) was expanded and the intensity of **(G&I)** CD31^+^ and **(H&J)** HLA-A^+^ staining across the circumference of the tumour vasculature was quantified. Numbers refer to the spatial positioning on the circumference of the tumour vasculature. Hoechst was used for nuclear staining. **(K-N)** Quantitative analysis of the immunofluorescence staining images represented in (F). Endothelialised cells were defined as CD31^−^/HLA-A^+^ cells spatially incorporated into the tumour vasculature. An endothelialised vessel was considered any blood vessel with the presence of one or more endothelialised cells. Samples from four mice (n=4) were analysed for each group. Results represent mean ± SEM. **p < 0.01, ****p < 0.0001.

Our results established that glioblastoma cells undergo endothelialisation in response to TGF-β-Smad1/5 signaling activation. We therefore sought to investigate the endothelialisation potential of two contrasting cell lines *in vivo* by orthotopically implanting luciferase-labelled U87MG or MU20 cells in immunocompromised mice. U87MG cells represent a model for increased endothelialisation potential in response to TGF-β-Smad1/5 signaling activation, while MU20 cells show limited phenotypic plasticity due to weak response to TGF-β. Quantification of the bioluminescence constitutively emitted by glioblastoma cell lines was used to monitor tumor growth and revealed that MU20 tumours grew faster and were comparatively larger in size than U87MG tumours by experimental endpoint (Fig. 4A-4B). Despite the difference in growth rates, mouse body weights remained similar until experimental endpoint (Fig. 4C). Interestingly, U87MG tumours displayed expansive growth with no clear invasive margins while MU20 tumours exhibited extensive invasion at the primary tumour margin (Fig. 4D).

Immunofluorescence analysis of U87MG tumours revealed human HLA-A staining around and within close proximity to CD31^+^ blood vessels (Fig. 4E). As expected, CD31^+^ blood vessels were found extensively throughout the primary tumour, characterised by dilated and irregular sized vessels (Fig. 4E). Noteworthy, these blood vessels also displayed many CD31^−^ cells (Fig. 4E), suggesting lining abnormalities. Close examination of tumor vasculature identified HLA-A^+^ cells lining blood vessels where CD31^+^ cells would be expected (Fig. 4E). We also quantified the pixel intensity of CD31 and HLA-A staining across the blood vessel circumference and identified a clear absence of CD31 staining from numbers 2-3 (zoomed image). This region was replaced by HLA-A^+^ staining, suggesting a change in the markers expressed within this part of the blood vessel (Fig. 4E-4G). CD31^+^ staining was also observed to be highly variable in intensity across blood vessels, confirming the irregularity of these blood vessels (Fig. 4E-4G). In contrast, MU20 tumours displayed relatively few and smaller blood vessels (Fig. 4E). HLA-A staining was still extensively observed across the tumour section. However, CD31^+^ blood vessels were less variable in intensity (Fig. 4, Fig. 4-4I). Noteworthy, HLA-A^+^ staining was not found within the blood vessel circumference (Fig. 4, Fig. 4-4I).

Moreover, MU20 tumours presented reduced number of blood vessels per field and these blood vessels were not endothelialised by tumor cells (Fig. 4J-4K). In turn, endothelialised glioblastoma cells were detected in approximately 14 % of the blood vessels in U87MG tumours, representing 33 % of all the cells forming such endothelialised vessels (Fig. 4L-4M).

### *In vivo* inhibition of Smad1/5 signalling reduces glioblastoma endothelialisation and whole brain tumour dissemination

**Fig. 5:**
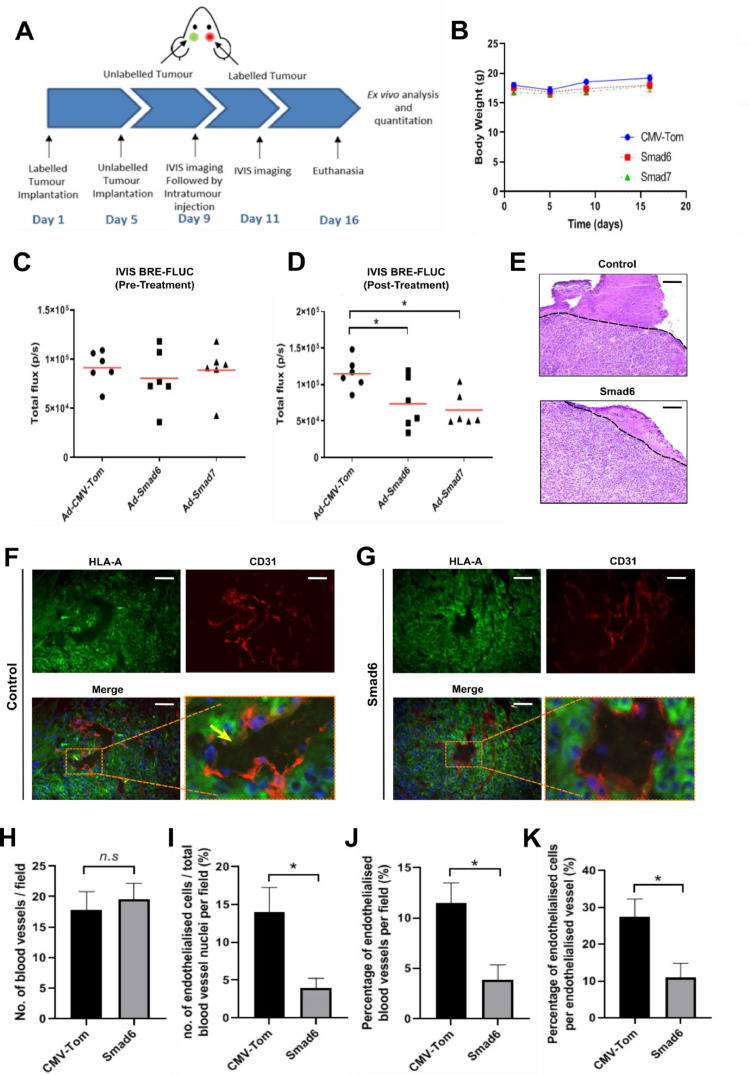
***In vivo* inhibition of Smad1/5 signalling reduces glioblastoma endothelialisation. (A)** Schematic illustration of the orthotopic contralateral xenograft mouse model used in this study. **(B)** Body weights of mice by treatment group. Day 1 corresponds to experimental start date with tumour implantation. IVIS bioluminescence imaging capture **(C)** before intracranial treatment **(D)** and 2 days after intracranial treatment. Six mice (n=6) were analysed in each group. **(E)** Representative H&E staining of U87MG-FLUC tumours. Scale bar: 50 μm. Smad7 treated tumours could not be observed at experimental endpoint and images for these group were not included. **(F-G)** Representative images of immunofluorescence staining analysis of **(F)** control and **(G)** Smad6-treated tumour sections. Arrows indicate CD31^−^/HLA-A^+^ vasculature staining. Scale bar: 50 μm. **(H-K)** Quantification of immunofluorescence staining for xenograft tumours. Endothelialised cells were defined as CD31^−^/HLA-A^+^ cells spatially incorporated into the tumour vasculature. An endothelialised vessel was considered any blood vessel with the presence of one or more endothelialised cells. Samples from four mice (n=4) were analysed for each group. Results represent mean ± SEM. ns: statistically non-significant, *p < 0.05.

To determine if a therapeutic strategy targeting Smad1/5 signalling could be used to suppress glioblastoma endothelialisation *in vivo*, we took advantage of our previously described glioblastoma model [34]. U87MG cells simultaneously labelled with BRE-firefly luciferase and CMV-Gaussia luciferase were orthotopically implanted into one hemisphere, while unlabelled U87MG cells were contralaterally implanted into the other hemisphere (Fig. 5A). At day 9 post-tumour implantation, mice were intracranially treated with either control adenovirus (*Ad-CMV-Tom*) or adenoviruses targeting TGF-β signaling activity (*Ad-Smad6* or *Ad-Smad7*). The body weights of all mice did not significantly change between treatment groups (Fig. 5B). Remarkably, however, while *in vivo* BRE-luciferase reporter activity was similar across all groups prior to treatment (Fig. 5C), a significant decrease in BRE-luciferase reporter activity was detected 2-days following *Ad-Smad6* or *Ad-Smad7* treatment (Fig. 5D).

Histologically, *Ad-CMV-Tom*- and *Ad-Smad6*-treated mice displayed similar tumor growth and morphological pattern (Fig. 5E). Tumours were not observed in the *Ad-Smad7*-treated group by histological analysis and sections are not shown. Immunofluorescence staining analysis of tumours treated with *Ad-CMV-Tom* demonstrated expansive and dense HLA-A^+^ tumour cells close to CD31^+^ blood vessels (Fig. 5F). Blood vessels displayed variable CD31 expression intensity and presented cells that were negative for CD31 (Fig. 5F). Close inspection of these cells confirmed HLA-A^+^ tumour cells lining the tumour vasculature (Fig. 5F). Interestingly, tumours treated with *Ad-Smad6* also exhibited dense and expansive HLA-A^+^ tumour cells, which closely surrounded CD31^+^ blood vessels (Fig. 5G). Nonetheless, in contrast to *Ad-CMV-Tom*-treated tumours, *Ad-Smad6*-treated tumours presented less variable CD31^+^ expression intensity across blood vessels and showed fewer (smaller) CD31 marker gaps (Fig. 5G). Moreover, although both *Ad-CMV-Tom* and *Ad-Smad6* treated tumour exhibited a similar number of blood vessels per field, the percentage of blood vessels with endothelialised cells decreased nearly 3-fold in the Smad6-treated group when compared with controls (Fig. 5H-5I). The average number of endothelialised cells observed per field decreased over 3.5-fold and the percentage of endothelialised cells within endothelialised blood vessels was also reduced in approximately 2.5-fold (Fig. 5J-5K).

**Fig. 6:**
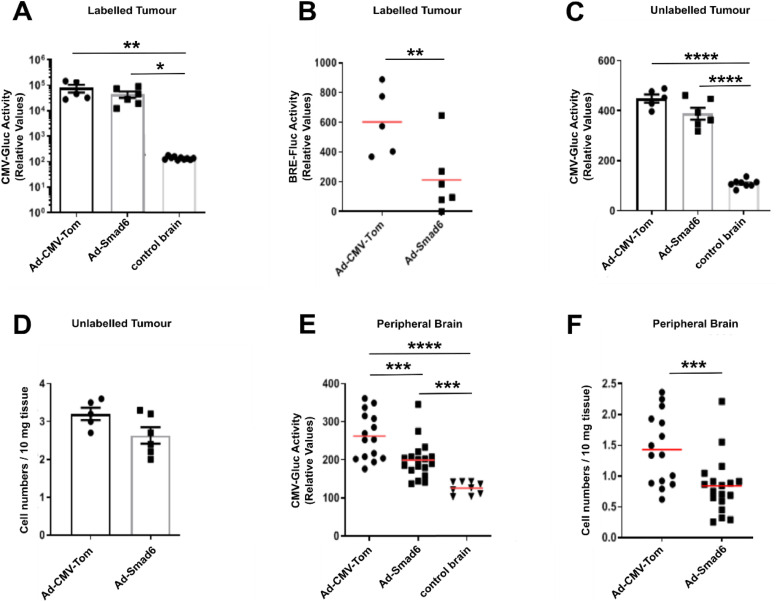
**Smad6-mediated *in vivo* Smad1/5 signalling inhibition impairs glioblastoma dissemination.***Ex vivo* luciferase assay was used to quantify Smad1/5 transcriptional levels (BRE-FLUC activity) and glioblastoma cell numbers (CMV-GLUC activity). Samples harvested from NOD-SCID mice orthotopically implanted with luciferase-labelled and unlabelled glioblastoma cells were homogenised and 10 mg tissue was used for *ex vivo* luciferase assays. Five (n=5) mice were analysed for the *Ad-CMV-Tom* group, and six (n=6) mice were analysed for the *Ad-Smad6* group. **(A)** Gaussia luciferase activity and **(B)** Firefly luciferase activity quantified in labelled tumour samples. **(C-D)** Tumour self-seeding was analysed by **(C)** quantifying the Gaussia luciferase activity in unlabelled tumour samples and **(D)** converting obtained results in corresponding cell numbers. **(E-F)** Similar procedure was used to analyse the dissemination of glioblastoma cells throughout the peripheral brain but using three independent brain samples from each mouse. Data represents mean ± SEM. *p < 0.05, **p < 0.01, ***p < 0.001, ****p < 0.0001.

Having established TGF-β-Smad1/5 signaling as the key molecular mechanism driving endothelialisation in glioblastoma, we next sought to define its functional relevance for tumour progression. Since tumour recurrence remains the most significant barrier to glioblastoma treatment [2,35], we utilised methods previously described by our group to analyse the overall dissemination and infiltration of the mouse brain as a model for tumour recurrence [34]. *Ex vivo* analysis of labelled primary tumours revealed similar Gaussia luciferase activity between *Ad-CMV-Tom* and *Ad-Smad6* groups, which were significantly higher than background control brain luciferase readings (Fig. 6A). Moreover, compared with tumors treated with *Ad-CMV-Tom*, reduced BRE luciferase transcriptional activity was also observed by *ex vivo* quantification of Firefly luciferase activity in tumors treated with *Ad-Smad6* (Fig. 6B), confirming sustained inhibitory effects until the experimental endpoint. Strikingly, analysis of unlabelled contralateral tumours also revealed positive Gaussia luciferase signal for all treatment groups (Fig. 6C). These results reinforce our previous findings by demonstrating that glioblastomas undergo tumour self-seeding [34], a phenomenon previously associated with cancer progression and aggressiveness [27,30,36] Calculation of the labelled numbers detected in the unlabelled tumours by using our previously described method [34] found 3.2 ± 0.2 cells/10 mg sample in *Ad-CMV-Tom*-treated mice and 2.6 ± 0.2 cells/10 mg sample in *Ad-Smad6*-treated mice, potentially representing a slight reduction in tumor self-seeding, although this possible difference was not statistically significant (Fig. 6D).. Finally, compared with background Gaussia luciferase signaling detected in control brain samples, significantly higher tumour specific Gaussia luciferase activity was also quantified in peripheral brain samples of all tumour-implanted mice (Fig. 6E). Yet, the number of Gaussia luciferase-labelled cells present in the peripheral brain were modestly, but significantly, reduced in *Ad-Smad6*-treated mice compared with *Ad-CMV-Tom* (Fig. 6F).

Altogether, these results demonstrate that glioblastoma progression is driven by the integration of tumour cells in the vasculature, which relies on the capacity of cancer cells to undergo endothelialisation in response to TGF-β-Smad1/5 signaling activation. Thus, targeting Smad1/5 activity may be a novel and effective therapeutic strategy to ameliorate glioblastoma progression as demonstrated in this study via treatment with *Ad-Smad6* (Fig. S7).

## Discussion

Glioblastoma progression and recurrence are critically associated with poor prognosis [1,2]. Despite extensive research for the characterization of glioblastoma biology, the key drivers of disease progression and recurrence are still poorly understood [2,3,14]. Accordingly, clinical trials evaluating therapeutic strategies for the treatment of glioblastoma patients have often relied on targeted therapies that were originally developed to manage diseases other than glioblastomas [3]. This observation helps to explain why advances in the treatment of glioblastoma patients are limited to marginal improvement, while simultaneously highlighting the urgent need for novel targeted therapies focused on glioblastoma's unique characteristics [4,35]. Here, we establish that TGF-β-ALK1-Smad1/5 signalling is a key driver of glioblastoma progression by promoting tumour cell endothelialisation. We report that TGF-β-mediated Smad1/5 activation in glioblastoma cells regulates tumour cell transdifferentiation into an endothelialised phenotype. Significantly, through discovery of this molecular mechanism we have been able to establish a novel mode of tumour dissemination contributing to widespread brain infiltration in glioblastoma (Fig. S7). Furthermore, we establish an unprecedented therapeutic approach that is designed to inhibit endothelialisation-mediated glioblastoma progression via exogenous Smad6 expression.

The infiltration and dissemination of glioblastoma tumour cells lead to glioblastoma progression [37]. These processes have often been associated with subpopulations of glioblastoma tumour cells that migrate into the dense neuropil by mimicking neuronal patterns [38,39]. However, an hematogenous route could also be relevant for the infiltration of glioblastoma cells throughout the brain. This is supported by our previous study where glioblastoma cells were detected in brain blood samples harvested from mice orthotopically implanted with U87MG cells [34]. Here, results demonstrated that U87MG cells are indeed highly dependent of endothelialisation to disseminate to the peripheral brain. Our findings agree with previous studies that have described an association between vasculogenic mimicry and late-stage/aggressive cancer progression and metastasis [[40], [41], [42]]. Nevertheless, this is the first time that glioblastoma tumour cells are demonstrated to integrate into the vasculature to actively participate in tumour dissemination.

Interestingly, although vasculogenic mimicry was firstly demonstrated over twenty years ago and its occurrence has been repeatedly correlated with poor prognosis for cancer patients, molecular mechanisms regulating this process have remained poorly characterized [43]. In this study, we show that TGF-β signaling is expanded in glioblastoma tumour cells and plays a critical role in vasculogenic mimicry by regulating their phenotype. TGF-β-ALK1-Smad1/5 signaling activation drives the transdifferentiation of glioblastoma tumour cells into endothelial-like cells, an outcome observed here in experimental models and validated in human patient sections. Previously, TGF-β signalling activity has been found to be upregulated in perivascular niches and closely associated with stemness markers such as Id1, CD44, Sox4 and Sox2, suggesting an underlying inherent plasticity of glioblastoma tumour cells [44,45]. The endothelialisation of glioblastoma tumour cells, however, requires the expansion of the TGF-β signalling for the activation of the intracellular effectors Smad1/5 downstream ALK1. Activation of TGF-β-ALK1-Smad1/5 signalling is known to regulate the growth and expansion of the vasculature, but it was never observed in cell types other than endothelial cells or associated with processes other than angiogenesis [[16], [17], [18],^33^].

Reinforcing the relevance of the TGF-β signalling for malignant tumours, inhibition of this molecular pathway has been shown to prevent the progression of many types of cancers in mouse models by reducing the phenotypic plasticity of cancer cells [27,30]. Similarly, our results showed that targeting TGF-β-Smad1/5 signaling activity impaired the endothelialisation of glioblastoma tumour cells. Specifically, we designed a therapeutic strategy based on the administration of *Ad-Smad6* adenovirus to induce exogenous expression of the specific inhibitor Smad6. TGF-β signaling is known to play a dual role in cancer by regulating cancer cell survival in early-stage cancers and enhancing the invasiveness of late-stage counterparts [13,18]. In agreement with TGF-β’s described contribution to highly aggressive glioblastomas, treatment with *Ad-Smad6*-inhibited TGF-β signaling did not impact glioblastoma tumor size. Importantly, however, our approach reduced lattice formation *in vitro* and decreased incorporation of tumour cells into the tumour vasculature *in vivo* to hinder glioblastoma systemic brain dissemination. Therefore, our findings not only establish a previously unknown mechanism responsible for glioblastoma progression as it also opens an avenue for the development of therapies capable of targeting the bona fide driver of glioblastoma progression.

Altogether, this study identifies endothelialisation as a novel part of the glioblastoma biology critically responsible for systemic brain tumour infiltration. Additionally, we describe TGF-β-ALK1-Smad1/5 signaling as the main mechanism regulating the phenotypic plasticity of glioblastoma cells, demonstrating an innovative and effective strategy to target this molecular pathway and impair glioblastoma progression. These findings provide a new explanation for the aggressive and incurable nature of glioblastoma that must be pursued as a therapeutic target to combat tumour progression and potentially ameliorate glioblastoma recurrence.

## Star methods

### Cells and cell culture

Human glioblastoma cell lines MU20 and MU41 were derived from two patients with pathologically confirmed glioblastoma at the Royal Melbourne Hospital (Parkville, Victoria, Australia). MU20 and MU41 cells were originally generated and cultured in serum-free conditions as neurospheres in non-adherent tissue culture plates and subsequently modified to adherent cells grown in monolayer by disassociating spheroid cultures and seeding cells onto adherent plates. All experiments outlined here describe the use of MU20 and MU41 cells as adherent cell lines below passage ten. Use of these cell lines in the laboratory was approved by the Melbourne Health Human Research and Ethics Committee (HREC 2012.219). U87MG obtained from the American Type Culture Collection (ATCC). All cells were maintained in Dulbecco's modified Eagle's medium and contained 10 % foetal bovine serum (FBS) (Thermo Fischer Scientific, USA), 2mM glutamime, 100U/ml penicillin and 100μl/ml streptomycin (Invitrogen, USA). Cells were incubated in a humidified atmosphere of 10 % CO_2_ 37^◦^C.

### Antibodies

Goat polyclonal antibodies anti-Smad6 (sc6034), anti-Smad7 (sc7004) and anti-Smad5 (sc7443) were purchased from Santa Cruz Biotechnology. Mouse monoclonal anti-Smad2/3 (S66220) antibody was purchased from Transduction Laboratories. Rabbit polycloncal anti-phospho-Smad2/3 and anto-phospho-Smad1/5 antibody was gifted from Prof. Peter Ten Dijke (Leiden University Medical Center, Netherlands). Monoclonal mouse anti-actin antibody was purchased from Sigma-Aldrich (A-4700). All primary antibodies used in this study were diluted 1:1000. HRP-conjugated secondary antibodies were purchased from Bio-Rad and diluted 1:5000 before use.

### Small molecule inhibitors, adenoviruses and cell treatment

Human recombinant TGFβ1 (100-21-100) and human recombinant BMP7 (120-03-10) were purchased from PeproTech and used at concentrations indicated throughout the study. The small molecule SB431542 (S4317, Sigma-Aldrich) was used as TGF-β signaling inhibitor based on its confirmed specificity for the kinase activity of TGF-β type 1 receptor. Adenovirus vectors *Ad-CMV-Td-Tomato* (CMV-Tomato), *Ad-BRE-Td-Tomato* (BRE-Tomado), *Ad-BRE-Firefly-Luciferase* (BRE-Fluc), *Ad-CAGA-Gaussia-Luciferase* (CAGA-Gluc), *Ad-CMV-Smad6* (Smad6) and *Ad-CMV-Smad7* (Smad7) were all generated in our laboratory. FuGENE® HD transfection reagent was purchased from Promega (E2312).

### Western blotting analysis

Cells were lysed in lysis buffer (30 mM HEPES, 1 % Triton-X-100, 2 mM MgCl, 150 mM NaCl, 5 mM EDTA) supplemented with protease inhibitor cocktail (Roche, Switzerland) on ice for 30 min. Lysates were centrifuged (13000 rpm; 15 min) and supernatant extracted. Lysates were mixed with Laemmli sample buffer (Bio-Rad Laboratories, Australia) and heated (95 ^◦^C; 10 min). Proteins were separated by SDS-PAGE and transferred to nitrocellulose membranes (GE Healthcare, Australia). After blocking with 4 % skim milk (1 h; RT), membranes were probed. The signal was developed using Western Lightning® ECL Pro Enhanced Chemiluminescence Substrate (PerkinElmer, USA). All western blotting results were representative of three separate experiments.

### Adenovirus fluorescence and immunofluorescence

Cells were transduced with *Ad-CMV-Td-Tomato* adenovirus in 5 % FBS DMEM media. Optimal expression was provided after 48 h infection. For *in* vitro immunofluorescence cells were fixed in 3.7 % paraformaldehyde (Sigma-Aldrich, USA) for 5-7 min and blocked in 1 % BSA (1 h; RT). Cells were probed with primary and ALEXA fluorescence secondary antibodies. Fluorescence signal was detected using a Leica fluorescent microscope. For tissue section immunofluorescence, fresh frozen tissue was sectioned at 6 microns and fixed in 100 % acetone a(-20 ^◦^C; 10 min). Sections were blocked in 5 % normal serum and probed with primary and ALEXA secondary antibodies. Slides were stained with 1 µg/µL Hoescht (5 min). Sections are mounted using VectaShield Antifade Mounting Media for Fluorescence (Vector Laboratories, USA) and sealed using clear nail polish. Fluorescent signal was detected using Leica fluorescent microscope and Leica sp5 Confocal microscope.

### Luciferase assay

Reporter cells (5000 cells/well in 96-well plate) were seeded and transduced with *Ad-BRE-Firefly-Luciferase* virus (MOI: 250) and *Ad-pCAGA-Gaussia-Luciferase* virus (MOI: 100) in 5 % FBS DMEM media. After 24 h infection, cells were serum starved in 100 μL serum free DMEM media for 16 h. Reporter cells were stimulated with indicated ligand (TGF-β or BMP7) for a further 6 h. Thereafter, cells were lysed and assessed for luciferase activity using Luciferase Assay Kit (Promega, USA) following the manufacturer's instructions. Luciferase activity was presented as the fold change as compared with control.

### *In vitro* lattice formation assay

48-well tissue culture plate were coated with Matrigel Basement Membrane Matrix (100 µL/well, BD Bioscience, Australia), which was polymerised at 37 ^º^C for 1 h. Cells were resuspended and seeded on matrigel at 4.5×10 [4] cells/mL in 5 % FCS DMEM media and incubated (6 h; 37 ^º^C). To investigate the effects of inhibitory Smad proteins on lattice formation, cells were transduced with *Ad-CMV-Td-Tomato, Ad-Smad6* or *Ad-Smad7* 48 h prior to seeding. Cultures were photographed using a Leica fluorescence microscope. Images were quantified using angiogenesis analyzer (http://image.bio.methods.free.fr/ImageJ/?Angiogenesis-Analyzer-for-ImageJ).

### Stable transfection of Firefly luciferase and Gaussia luciferase

U87MG cells were stably transfected with pGL4.51[luc2/CMV/Neo] (Promega, NSW, Australia) or pCMV-Gluc-KDEL (Prolume Ltd., USA) vector using FuGENE® HD (Promega, Australia). Cells underwent selection with 2 mg/mL G418 (Roche, Switzerland) and stable clonal colonies obtained.

### *In vivo* experiments

Animal experiments were approved by the University of Melbourne Animal Ethics Committee (1614055). Six-to-eight-week-old female NOD-SCID mice were purchased from Animal Resource Centre (ARC) Canning Vale, Western Australia and housed in the Department of Surgery Animal Facility, University of Melbourne, Victoria. For intracranial implantation, mice were anaesthetized with 4 % isoflurane and maintained at 2 % isoflurane for the procedure. Mice were shaved and positioned on the stereotactic. A small burr hole < 1 mm diameter was fashioned and 5×10 [4] firefly-labelled cells implanted in 5 μL using a 27-gauge needle at a flow rate of 1 μL/min. The needle was retracted and the hole sealed using sterile bone wax. Mice were monitored for recovery and 5 days later a non-labelled contralateral tumour was implanted using the same procedure. Once recovered mice were transferred to Monash Institute of Pharmaceutical Sciences for whole-body imaging using an *In Vivo* Imaging System (IVIS) and blood collection. One hundred and fifty milligrams per kilogram D-luciferin was intraperitoneally injected into mice for IVIS Illumina II (Caliper Life Sciences, USA) bioluminescence detection. Following experimental endpoint, mice were euthanized and brains harvested. Firefly-labelled tumours were frozen in optimal cutting temperature (OCT) and sectioned for immunofluorescence staining. Unlabeled tumours were lysed in cell culture lysis buffer (Promega, USA) and luciferase bioluminescence quantified on a luminometer.

### Human glioblastoma images

Human glioblastoma images of *in situ* hybridization and hematoxylin and eosin (H&E) tissue sections were obtained from the publicly available Ivy Glioblastoma Atlas Project (Ivy GAP) (https://glioblastoma.alleninstitute.org/).

## Statistical analysis

All experiments were performed three times. The data were recorded as mean ± standard error of the mean (SEM) and statistical analysis was performed using Graphpad Prism 6 (GraphPad Software Inc, USA; v6.01). Differences between groups were analysed by one-way ANOVA with Tukey's multi-comparisons test or using the student's t test. Only two-sided tests were used in this study. p-value < 0.05 was considered statistically significant.

## CRediT authorship contribution statement

**Thomas M.B. Ware:** Methodology, Investigation, Data curation. **Adilson Fonseca Teixeira:** Writing – review & editing, Validation, Data curation. **Josephine Iaria:** Methodology. **Rodney B. Luwor:** Project administration. **Hong-Jian Zhu:** Writing – review & editing, Writing – original draft, Supervision, Resources, Project administration, Methodology, Investigation, Funding acquisition, Formal analysis, Conceptualization.

## Declaration of competing interest

The authors declare that they have no known competing financial interests or personal relationships that could have appeared to influence the work reported in this paper.

## Appendix Supplementary materials

Image, application 1
