# Supplementary material for: Systemic brain dissemination of glioblastoma requires transdifferentiation into endothelial-like cells via TGF-β-ALK1-Smad1/5 signaling
Source: Neoplasia. 2024 Dec 25;60:101110. doi: 10.1016/j.neo.2024.101110 (PMC11732171; doi:10.1016/j.neo.2024.101110)
Supplement: Supplementary file 1 [file mmc1.doc]

**SUPPLEMENTARY INFORMATION**

**Systemic brain dissemination of glioblastoma requires transdifferentiation into endothelial-like cells via TGF-β-ALK1-Smad1/5 signaling**

Thomas M.B. Ware^1,2^; Adilson Fonseca Teixeira^1,2^; Josephine Iaria^1,2^; Rodney B. Luwor^1,2,3,4^ and Hong-Jian Zhu^1,2,5*^

^1^Department of Surgery, (The Royal Melbourne Hospital), The University of Melbourne, Parkville 3050, Victoria, Australia

^2^Huagene Institute, Kecheng Science and Technology Park, Pukou District, Nanjing 211806, Jiangsu, China

^3^Fiona Elsey Cancer Research Institute, Ballarat, Victoria 3350, Australia

^4^Federation University, Ballarat, Victoria 3350, Australia

^5^Lead contact

***Corresponding Author:**

Dr. Hong-Jian Zhu, Department of Surgery, The University of Melbourne, The Royal Melbourne Hospital, Parkville 3050, Victoria, Australia. Email [hongjian@unimelb.edu.au](mailto:hongjian@unimelb.edu.au)

**Supplementary Table**

**Table S1. Patients presenting gaps in the tumour vasculature.**

|  | *ESM1* | *MECOM* | *ENPEP* | *EPAS1* | *GFAP* |
| --- | --- | --- | --- | --- | --- |
| Patients with blood vessel gaps (%) | 86.4 | 87.5 | 73.3 | 60.0 | 100 |
| Number of Patients | 22 | 24 | 15 | 15 | 7 |

**SUPPLEMENTARY FIGURES**

**Fig. S1**

**
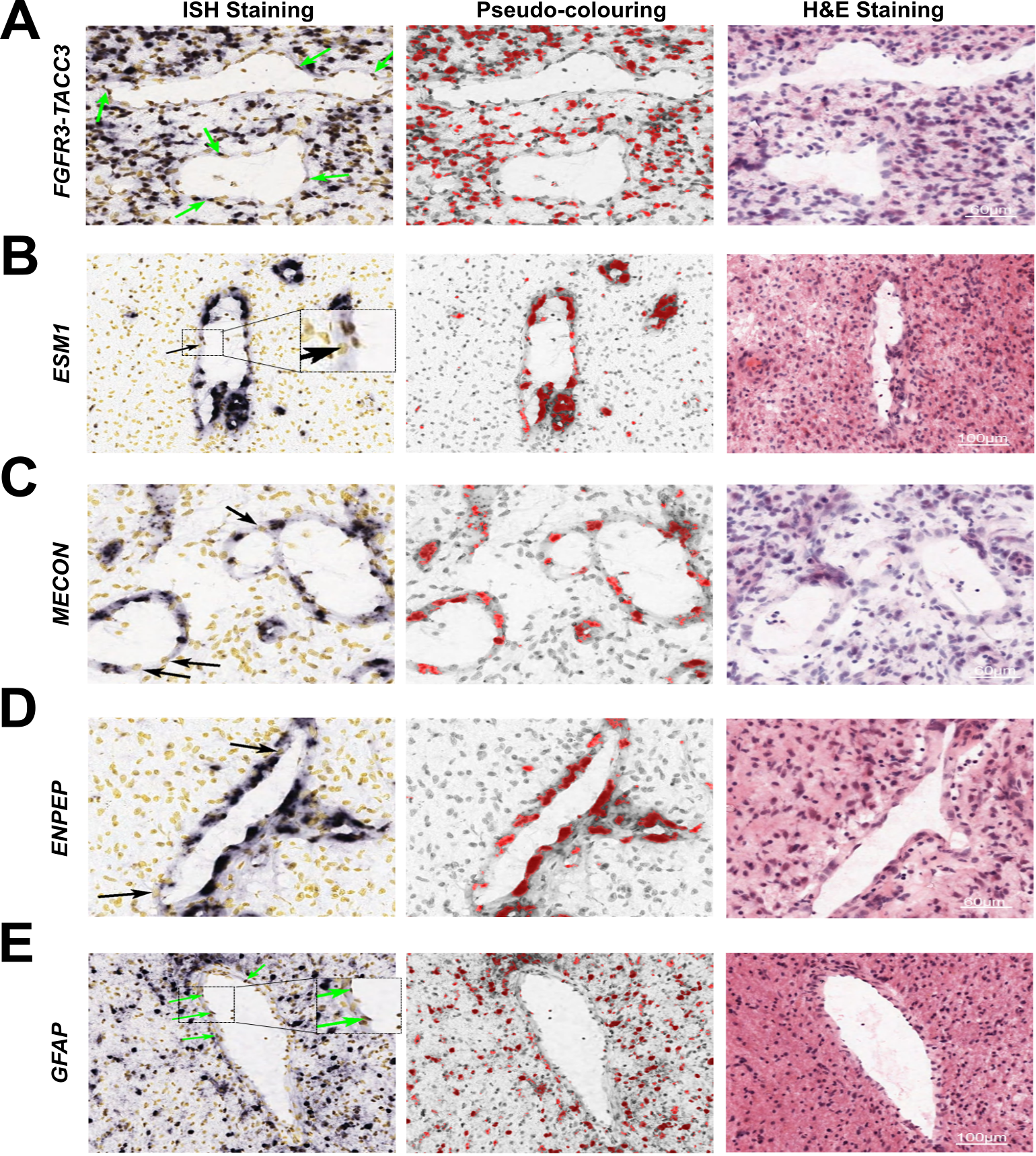
**

**Fig. S1. A second glioblastoma with *FGFR3-TACC3* fusion gene also presents with tumour cell incorporated into the vasculature endothelium, related to Figure 1.** *In situ* hybridisation (ISH) staining and H&E staining were obtained from the Ivy Glioblastoma Atlas Project (Ivy GAP). Images are representative of a single patient with confirmed *FGFR3-TACC3* fusion gene. Tumour samples containing blood vessels were stained for **(A)** *FGFR3-TACC3*, **(B)** *ESM1*, **(C)** *MECON*, **(D)** *ENPEP*, and **(E)** *GFAP*. Arrows in the left panels indicate positive staining within the endothelium. Middle panel shows the pseudo-colouring for cells positively stained for the target nucleic acid. Right panel shows the nearest H&E section.

**Fig. S2**

**
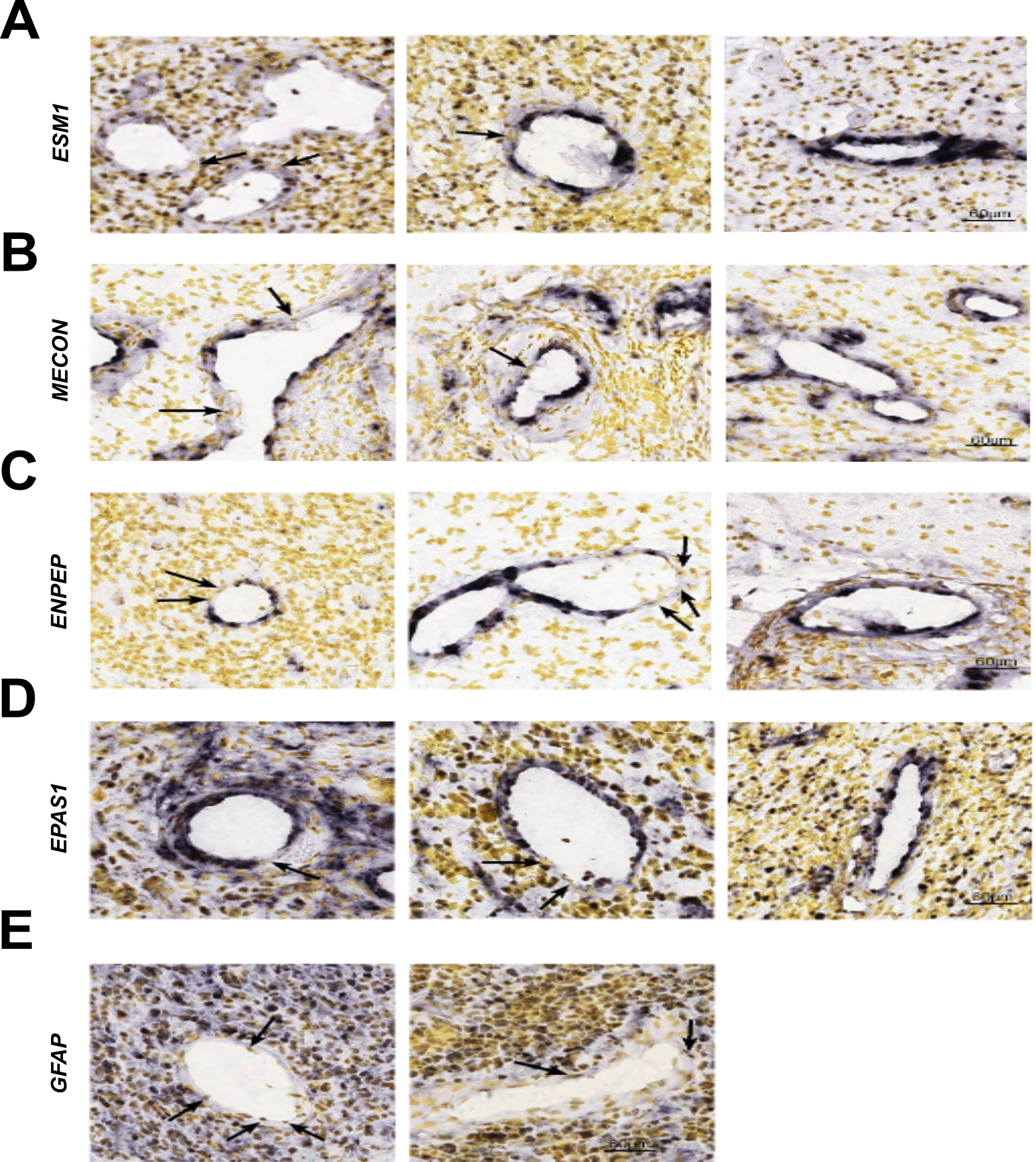
**

**Fig. S2. Glioblastoma patient samples exhibit non-continuous endothelial cell marker expression with non-endothelial cells incorporated into the endothelium of tumour vasculature, related to Figure 1.** *In situ* hybridisation (ISH) staining images were obtained from the Ivy Glioblastoma Atlas Project (Ivy GAP). Images are representative of multiple patients. Tumour samples containing blood vessels were stained for **(A)** *ESM1*, **(B)** *MECON*, **(C)** *ENPEP*, **(D)** *EPAS1*, and **(E)** *GFAP*. In (A-D), arrows indicate negative staining within the endothelium on left and middle panels, while highlighting continuous endothelial enriched ISH staining on the right panels. In (E), arrows indicate positive staining within the endothelium.

**Fig. S3**

**
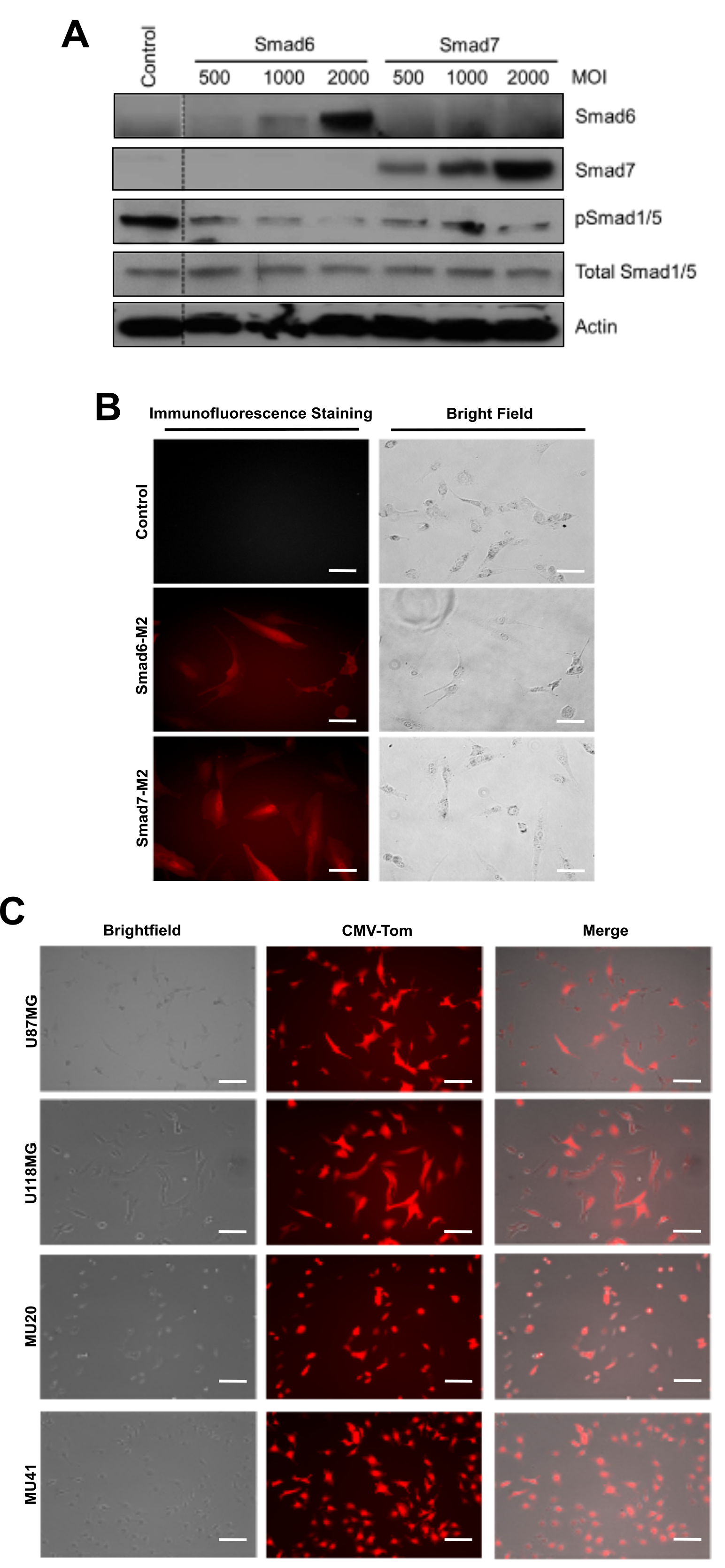
**

**Fig. S3. *Ad-Smad6* and *Ad-Smad7* are efficiently transduced in glioblastoma cells, related to Figure 2. (A)** Western blot analysis of U87MG cells transduced with *Ad-CMV-Td-Tom* (control adenovirus), *Ad-Smad6-M2* or *Ad-Smad7-M2* for 48 hours using indicated MOI. Exogenous Smad6 and Smad7 levels were analysed by using an anti-M2 antibody. Phosphorylated (p)Smad1/5 levels were analysed to evaluate Smad6 and Smad7 inhibitory activity. Total Smad1/5 and Actin levels were used as loading controls. **(B)** Immunofluorescence staining analysis of U87MG cells transduced as in (A). Scale bar: 100 μm. **(C)** Analysis of transduction efficiency by fluorescence microscopy. Glioblastoma cell cultures were analysed 48 hours after transduction with *Ad-CMV-Td-Tom* (MOI: 1000). Scale bar: 50 μm.

**Fig. S4**

**
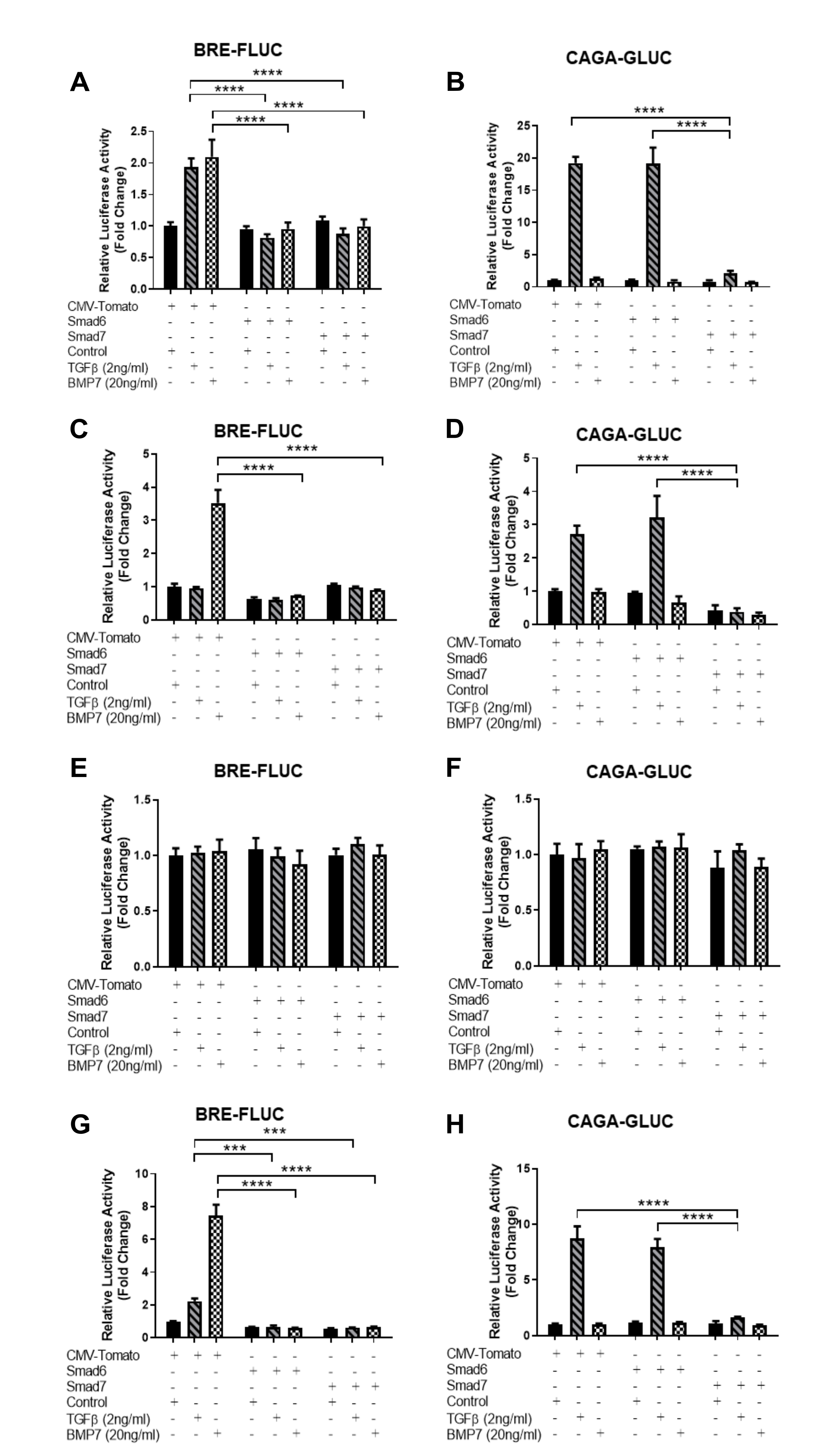
**

**Fig. S4. *Ad-Smad6* and *Ad-Smad7* transduction reduce Smad1/5 phosphorylation in glioblastoma cells, related to Figure 2.** Dual luciferase assay was used to quantify Smad1/5 transcriptional activity and Smad2/3 transcriptional activity in glioblastoma cells transduced with *Ad-BRE-FLUC* and *Ad-CAGA-GLUC*. Glioblastoma cells were additionally transduced ± *Ad-CMV-Td-Tom* (control adenovirus), *Ad-Smad6* or *Ad-Smad7* and treated ± 2 ng/mL TGF-β or 20 ng/mL BMP7 for 6 hours. Results show the normalized bioluminescence quantified in **(A-B)** U87MG, **(C-D)** U118MG, **(E-F)** MU20, and **(G-H)** MU41 cell lysates. Data represent mean ± SEM relative to three independent experiments (n=3). ***p < 0.001, ****p < 0.0001; One-way ANOVA with Tukey’s multiple comparisons test.

**Fig. S5**

**
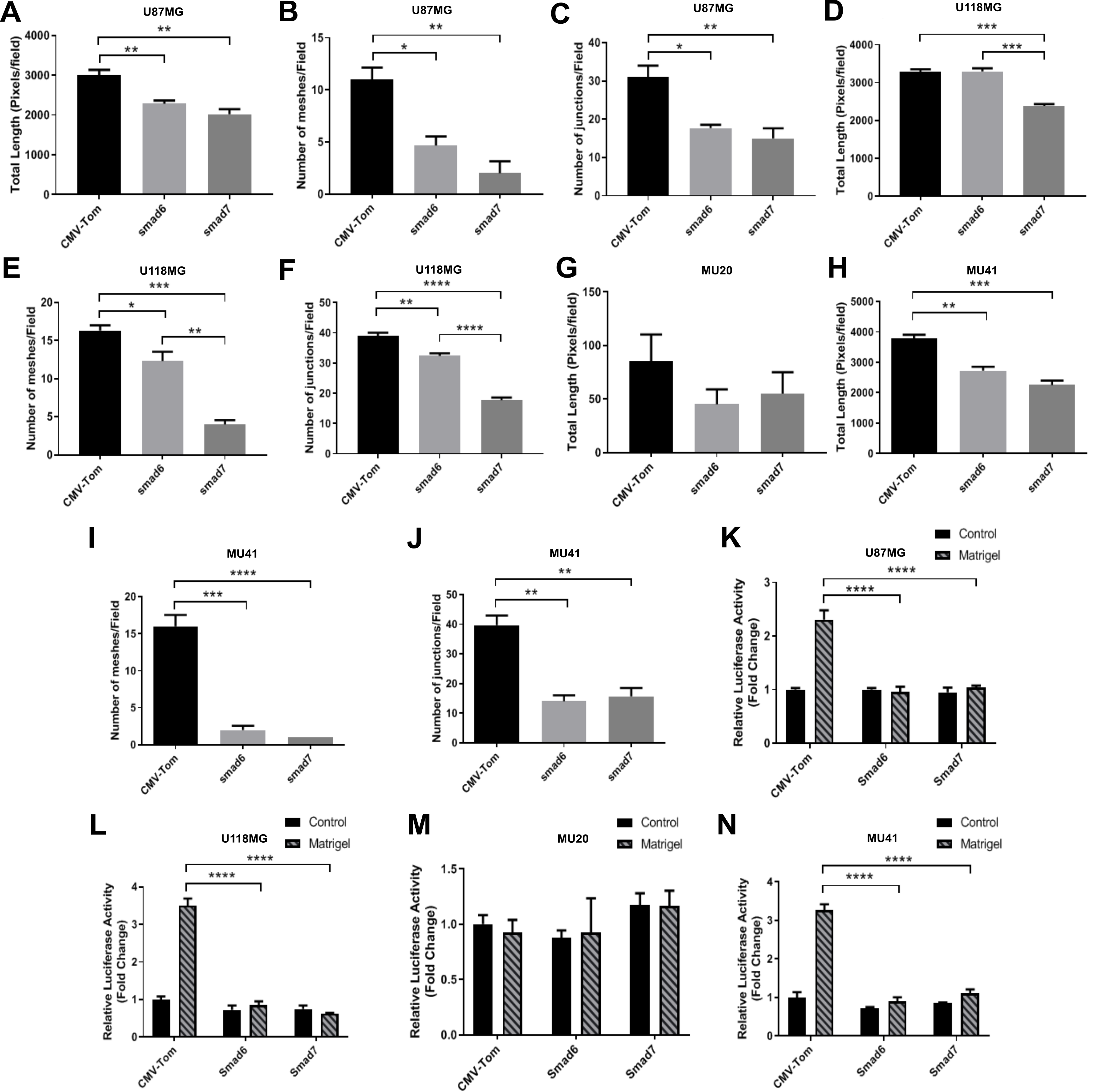
**

**Fig. S5. TGF-β-Smad1/5 signalling drives lattice formation in glioblastoma cell lines *in vitro*, related to Figure 2.** Glioblastoma cell lines transduced with *Ad-CMV-Tom* (control adenovirus), *Ad-Smad6* or *Ad-Smad7*. Forty-eight hours after transduction, cells were seeded onto uncoated or matrigel-coated wells and analysed 6 h post-seeding. **(A)** Number of junctions, **(B)** number of meshes, and **(C)** cell total length were quantified and normalized per field for U87MG cell cultures. The same parameters were quantified for **(D-F)** U118MG, **(G)** MU20, and **(H-J)** MU41 cell cultures. MU20 cell cultures did not form meshes or junctions and graphical representation of these parameters is omitted. **(K-N)** Quantification of Firefly luciferase activity in **(K)** U87MG, **(L)** U118MG**, (M)** MU20, and **(N)** MU41 cells transduced with BRE-FLUC following image acquisition of lattice formation. Results represent mean ± SEM relative to three independent experiments (n=3). *p < 0.05, **p < 0.01, ***p < 0.001, ****p < 0.0001; One-way ANOVA with Tukey’s multiple comparisons test.

**Fig. S6**

**
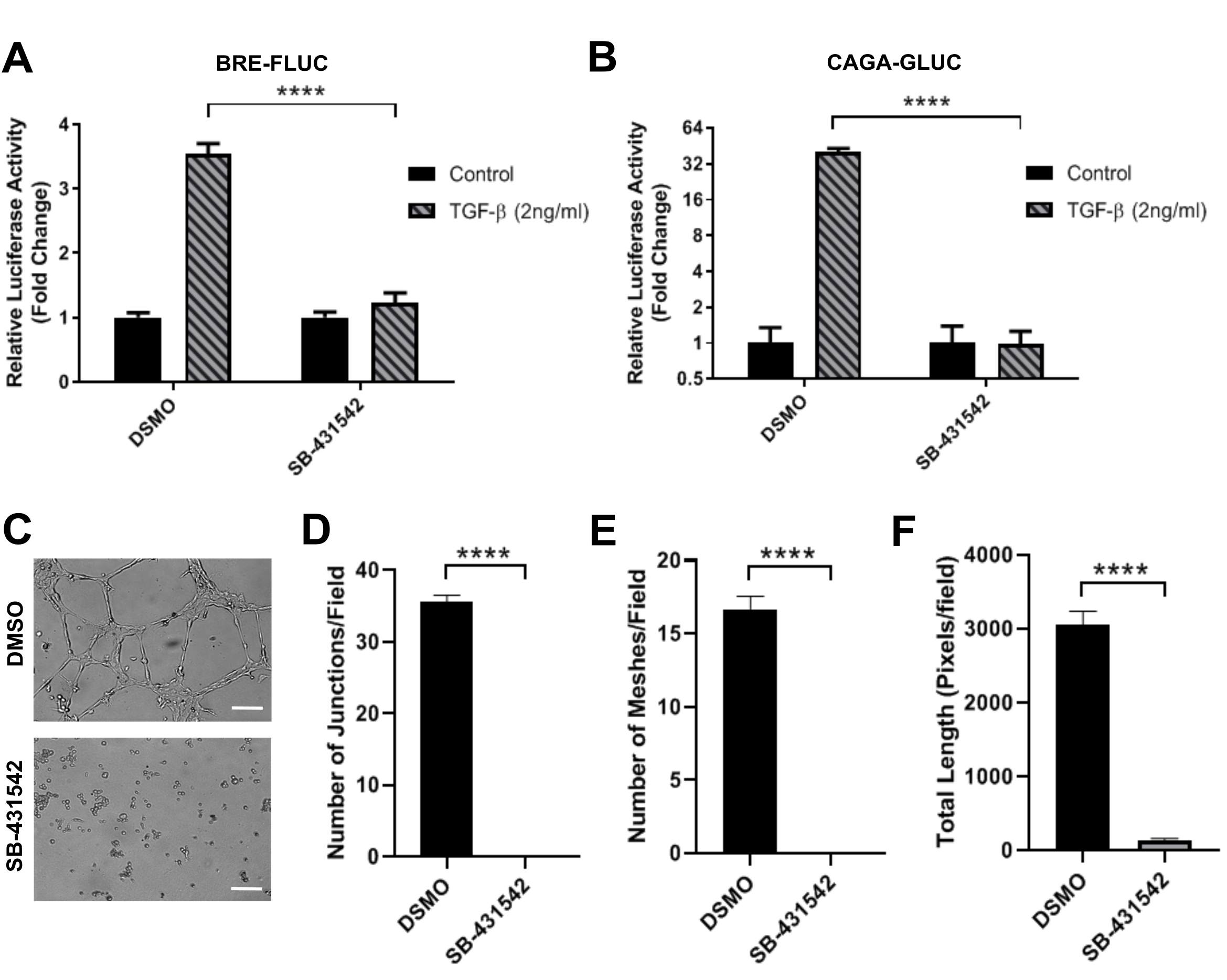
**

**Fig. S6. SB-431542 abolishes lattice formation in U87MG glioblastoma cells, related to Figure 3.** Dual luciferase assay was used to quantify **(A)** Smad1/5 transcriptional activity and **(B)** Smad2/3 transcriptional activity in U87MG glioblastoma cells transduced with *Ad-BRE-FLUC* and *Ad-CAGA-GLUC*. Cell cultures were serum starved and treated ± 2 ng/mL TGF-β along with either DSMO (vehicle) or 10 µM SB-431542 for 6 hours. **(C)** Morphological analysis of U87MG cells were seeded onto matrigel-coated wells and treated with DSMO (vehicle) or 10 µM SB-431542. Scale bar: 50 μm. Lattice-related parameters were quantified and represented as **(D)** number of junctions, **(E)** number of meshes, or **(F)** total cell length per field. Results represent mean ± SEM relative to three independent experiments (n=3). ****p < 0.0001; One-way ANOVA with Tukey’s multiple comparisons test.

**Fig. S7**

**
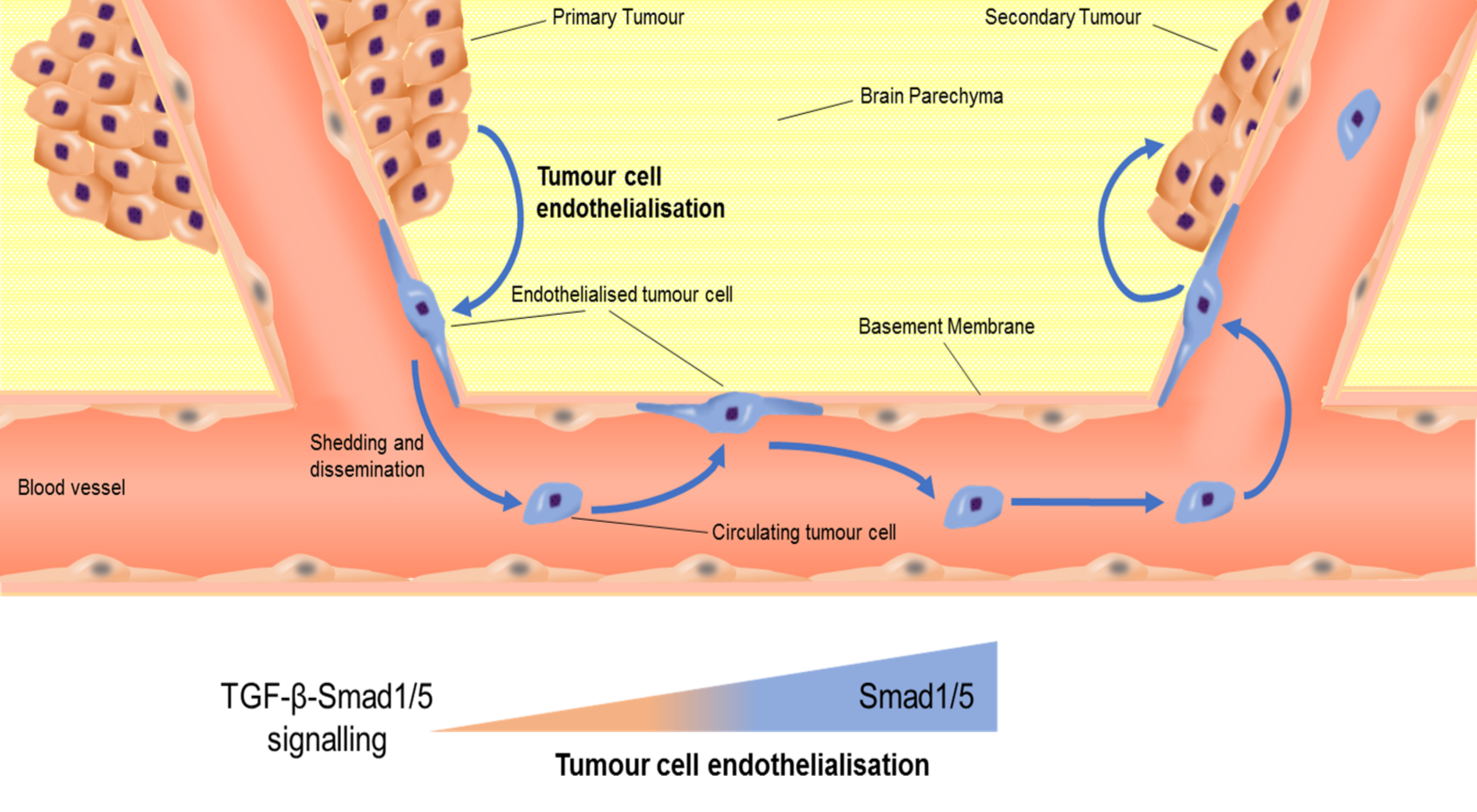
**

**Fig. S7. Working model.** Glioblastoma progression relies on tumour cells dissemination mediated by vasculogenic mimicry. Th is process is defined as the integration of glioblastoma tumour cells into the local vasculature and relies on phenotypic plasticity exhibited by cancer cells that is regulated by the TGF-β signaling. Specifically, TGF-β-ALK1-Smad1/5 signaling activation in glioblastoma tumour cells promotes endothelialisation; a phenotypic switching towards an endothelial-like phenotype. Endothelialised glioblastoma tumour cells integrate into the tumour vasculature through vascular mimicry to disseminate as circulating tumour cells to secondary sites within the peripheral brain. Consequently, endothelialisation-mediated tumour cell dissemination helps to explain the recurrence of glioblastomas and the poor overall survival of glioblastoma patients.
